# Endoscopic vs Open Surgeries for Piriformis Syndrome: A Systematic Review and Meta‐Analysis

**DOI:** 10.1155/aort/5126019

**Published:** 2026-07-30

**Authors:** Fanny Indra Warman, Jifaldi Afrian Maharaja Dinda Sedar, Muhammad Hanun Mahyuddin, Williem Harvey

**Affiliations:** ^1^ Department of Orthopaedic and Traumatology, Prof. Dr. R. Soeharso Orthopaedic Hospital, Surakarta, Indonesia; ^2^ Department of Orthopaedic and Traumatology, Faculty of Medicine, Universitas Airlangga, Surabaya, Indonesia, unair.ac.id; ^3^ Department of Orthopaedic and Traumatology, Dr. Soetomo General Academic Hospital, Surabaya, Indonesia, rsudrsoetomo.jatimprov.go.id

**Keywords:** decompression, endoscopy, open surgery, pain measurement, piriformis syndrome

## Abstract

**Background:**

Piriformis syndrome (PS) is a neuromuscular condition caused by compression or irritation of the sciatic nerve near the piriformis muscle, resulting in buttock pain that may radiate to the lower limb. Although most patients improve with conservative treatment, some develop refractory PS and require surgery. Endoscopic decompression has emerged as a minimally invasive alternative to open surgery. This study evaluated the available evidence on surgical outcomes for refractory PS.

**Methods:**

This systematic review and meta‐analysis followed PRISMA guidelines. PubMed, Scopus, Medline, and ScienceDirect were searched without year restriction. Because no direct comparative studies between endoscopic and open surgeries were identified, both approaches were analyzed separately. Eligible studies reported pain outcomes using the visual analog scale (VAS) and functional outcomes using the modified Harris hip score (mHHS).

**Results:**

Twelve studies met the inclusion criteria, including nine endoscopic and three open‐surgery studies. Nine studies were eligible for quantitative synthesis. Pooled analysis of six endoscopic studies showed a significant reduction in VAS (mean difference −4.7; 95% CI −5.2–−4.2) and improvement in mHHS (mean difference +25.6; 95% CI +16.0–+35.3). Endoscopic complication rates ranged from 0% to 12% and were mostly minor. Open‐surgery evidence was limited and synthesized narratively only.

**Conclusion:**

Endoscopic decompression appears to provide favorable outcomes in carefully selected patients with refractory PS. However, definitive superiority over open surgery cannot be established from the current literature.

## 1. Introduction

Piriformis syndrome (PS) is a neuromuscular condition that occurs when the sciatic nerve is compressed or irritated as it traverses or passes near the piriformis muscle in the deep gluteal region [1, 2]. Although it is responsible for approximately 6%–8% of all sciatica cases, PS is frequently underdiagnosed because its symptoms overlap with those of lumbar radiculopathy, sacroiliac joint dysfunction, sacroiliitis, and various hip disorders [3, 4]. This diagnostic challenge is particularly relevant in patients presenting with buttock pain or inflammatory back pain, in whom PS may mimic or be confused with sacroiliitis [5]. In a clinical setting, PS typically presents as buttock pain radiating along the posterior thigh. Furthermore, it is often worsened by prolonged sitting or specific hip movements, with positive findings on physical tests such as the Freiberg, Pace, or flexion‐adduction‐internal rotation (FAIR) maneuvers [6, 7]. The lack of standardized diagnostic criteria, combined with diverse etiological factors including trauma, anatomical variations, and occupational risks, contributes to persistent diagnostic uncertainty [8, 9].

Conservative interventions such as physical therapy, nonsteroidal anti‐inflammatory drugs (NSAIDs), corticosteroid or botulinum toxin injections, and lifestyle modifications remain the first‐line management for PS [1, 10]. While these approaches achieve symptom relief in most patients, up to 20%–30% still develop refractory PS. This is characterized by persistent pain even after at least 3 months of optimized nonoperative treatment [2, 11]. For these patients, surgical decompression of the sciatic nerve via piriformis tendon release or neurolysis becomes necessary [4, 7].

Traditionally, open surgery has been the standard approach for treating PS, offering direct visualization and decompression of the nerve [1, 8]. However, open procedures are sometimes associated with risks such as extensive tissue dissection, longer recovery times, postoperative adhesions, and potential neurovascular injury [3, 4]. In recent decades, endoscopic and arthroscopic techniques have emerged as minimally invasive alternatives, providing enhanced visualization of the deep gluteal space, reduced morbidity, quicker rehabilitation, and the ability to treat concomitant intra‐ and extrapelvic lesions [7, 10]. Numerous studies have reported significant improvements in pain, functional outcomes, and quality of life, often accompanied by low complication rate, upon receiving endoscopic decompression [6, 11, 12].

Nevertheless, direct comparisons between open and endoscopic approaches are difficult because of factors such as heterogeneity in diagnostic criteria, surgical techniques, patient selection, and outcome measures [8, 10]. Recent evidence suggests that while open surgery may still be the preferred treatment for complex cases with extensive fibrosis, endoscopic decompression offers comparable or even superior results regarding pain relief, functional recovery, and patient satisfaction [9, 13].

Considering that more and more people adopt these minimally invasive procedures and no consensus has been made on the optimal surgical strategy for PS, it is essential to perform a systematic review and meta‐analysis that directly compare endoscopic versus open surgeries. This study aims to (i) synthesize current evidence, (ii) evaluate clinical outcomes, complication rates, and functional improvements, and (iii) provide guidance for surgical decision‐making in the management of refractory PS.

## 2. Methods

This study was conducted as a systematic review and meta‐analysis in accordance with the Preferred Reporting Items for Systematic Reviews and Meta‐Analyses (PRISMA) guidelines. The study protocol was prospectively registered in the International Prospective Register of Systematic Reviews (PROSPERO) under registration number CRD420251156529.

A comprehensive literature search was carried out in PubMed, Scopus, Medline, and ScienceDirect without restriction on publication year. The search aimed to identify studies evaluating surgical treatment for PS, including both endoscopic and open approaches. No study directly comparing endoscopic and open surgery within the same study population was identified. Therefore, the two approaches were analyzed separately.

The search strategy was structured using two sets of Medical Subject Headings (MeSH) terms. The first set targeted endoscopic approaches and was defined as (“Piriformis Syndrome” [MeSH]) AND (“Endoscopy” OR “Arthroscopy” OR “Minimally Invasive Procedures”) AND (“Decompression” OR “Surgery”). The second set targeted open surgical approaches and was defined as (“Piriformis Syndrome” [MeSH]) AND (“Open Surgery” OR “Release” OR “Mini Open”) AND (“Neurolysis” OR “Decompression” OR “Tenotomy”). In addition, the reference lists of eligible studies were manually screened to identify any further relevant articles.

Studies were included if they (i) involved patients with PS undergoing either endoscopic or open surgical intervention, (ii) were original human studies, and (iii) reported quantitative outcome measures. Eligible study designs included retrospective or prospective observational studies, cohort studies, and clinical trials. Review articles, case reports, technical notes, conference abstracts, non‐English publications, articles without accessible full text, and studies without relevant outcome data were excluded.

Two reviewers independently screened the titles, abstracts, and full texts of all identified records. Any disagreements were resolved through discussion and consensus. Data extraction included author, publication year, study design, intervention type, sample size, and reported outcomes.

All studies meeting the inclusion criteria were included in the qualitative synthesis. However, only studies reporting preoperative and postoperative values of the visual analog scale (VAS) and/or modified Harris hip score (mHHS), together with sufficient statistical information for pooling, were included in the quantitative synthesis. Studies were excluded from meta‐analysis if they did not report pre‐ and post‐intervention data, did not provide measures of variance, used incompatible outcome scales, or reported outcomes in a manner unsuitable for pooled analysis. These studies were retained in the qualitative synthesis but excluded from the quantitative synthesis.

The methodological quality of the included studies was assessed using the Newcastle–Ottawa scale (NOS) for observational studies. Pooled analyses were conducted using a random‐effects model based on the DerSimonian–Laird method. Mean differences between preoperative and postoperative values were calculated for VAS and mHHS. Statistical heterogeneity was assessed using the *I* ^2^ statistic. Sensitivity analysis was performed by excluding outlier studies to evaluate the robustness of the findings. Funnel plots were visually inspected to explore potential publication bias.

**FIGURE 1:**
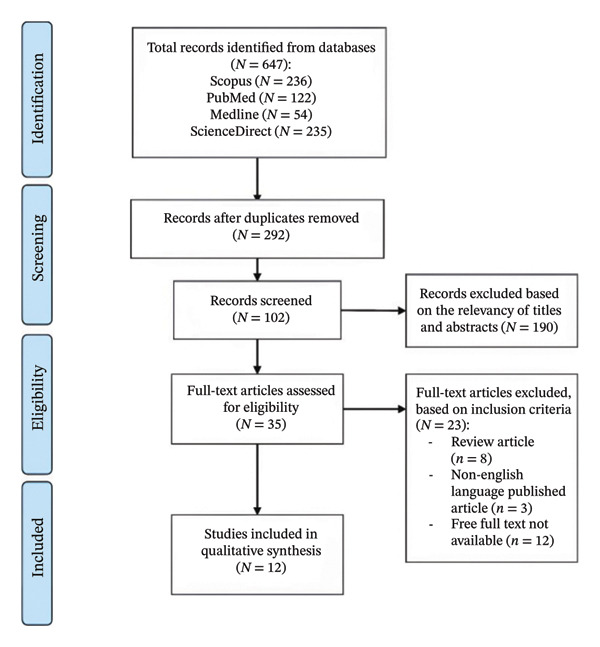
PRISMA flow diagram of study selection.

The study selection process is presented in Figure 1. A total of 647 records were identified from the four databases, including 236 from Scopus, 122 from PubMed, 235 from ScienceDirect, and 54 from Medline. After duplicate removal, 292 unique records remained. Following title and abstract screening, 35 full‐text articles were assessed for eligibility. Of these, 23 studies were excluded because they were review articles (*n* = 8) or non‐English publications (*n* = 3) or had no accessible full text (*n* = 12). Finally, 12 studies met the inclusion criteria and were included in the qualitative synthesis. These consisted of nine studies evaluating endoscopic techniques and three studies evaluating open surgery.

Of the 12 studies included in the qualitative synthesis, 9 studies met the criteria for quantitative synthesis (meta‐analysis). The remaining studies were excluded from meta‐analysis because they were single‐arm or case series studies and lacked an appropriate comparator structure for meta‐analysis.

## 3. Results

A total of 12 studies met the inclusion criteria and were included in the qualitative synthesis, comprising 9 studies on endoscopic procedures and 3 studies on open surgery for PS (Table 1). Among these, 9 studies were eligible for quantitative synthesis, including 8 endoscopic studies and 1 open‐surgery study. The remaining 3 studies were retained in the qualitative synthesis only because they were single‐arm or case series studies and/or did not provide an appropriate comparator structure for quantitative pooling. Across studies, the most frequently reported outcomes were pain intensity measured by the VAS and hip‐related function assessed by the mHHS, while other reported outcomes included the Benson score, WOMAC, iHOT‐12, and patient satisfaction.

**TABLE 1:** Study characteristics.

**No.**	**Author, year**	**Methods (study design)**	**Intervention vs comparison and total participants**	**Main outcome**
**Endoscopy treatment**				

1	Park et al., 2016 [12]	Retrospective observational cohort	Endoscopic sciatic nerve decompression; 60 patients (45 atrauma, 15 trauma)	● VAS ↓ 7.4 ⟶ 2.6 ● mHHS ↑ 81.7 ⟶ 91.8 ● Better outcomes in atrauma group

2	Sun et al., 2023 [10]	Retrospective observational	Arthroscopic sciatic neurolysis with ultrasound guidance; 30 patients	● VAS ↓ 5.0 ⟶ 0.5 ● mHHS ↑ 64 ⟶ 95 ● 90% good/excellent Benson score

3	Aguilera‐Bohorquez et al., 2018 [11]	Retrospective observational	Endoscopic sciatic nerve decompression; 41 patients (44 operations)	● WOMAC ↓ 63 ⟶ 26 ● Functional improvement ● 4 revision treatment procedures needed

4	Martin et al., 2011 [8]	Retrospective case series	Endoscopic decompression (fibrovascular band resection, piriformis/hamstring release); 35 patients	● VAS ↓ 6.9 ⟶ 2.4 ● mHHS ↑ 54.4 ⟶ 78 ● 83% no postoperative sit pain

5	Quesada‐Jimenez et al., 2025 [9]	Retrospective case series	Endoscopic piriformis release + sciatic neurolysis; 18 patients	● Significant improvements in mHHS, NAHS, HOS‐SSS, and VAS ● High patient satisfaction (8.3/10)

6	Ilizaliturri Jr et al., 2018 [6]	Case series	Endoscopic piriformis tendon release + sciatic nerve exploration; 15 patients	● mHHS ↑ 46.8 ⟶ 84.9 ● VAS ↓ 7.4 ⟶ 1.9 ● 93% good/excellent outcome

7	Vanermen and Van Melkebeek, 2021 [7]	Retrospective survey study	Endoscopic piriformis release + neurolysis; 45 patients	● VAS ↓ 7.4 ⟶ 1.9 ● 75% good/excellent Benson scale ● 3 wound infections

8	Park et al., 2025 [2]	Retrospective cohort (propensity‐matched)	Endoscopic piriformis release (EPR) vs pulsed radiofrequency (PRF); 230 patients (115 per group)	● At 6 mo: EPR NRS 2.2 vs PRF 2.9 (*p* = 0.018); EPR higher satisfaction but more complications

9	Parodi et al., 2023 [14]	Prospective observational	Endoscopic release of sciatic nerve (fibrovascular band resection, no piriformis tenotomy); 57 patients	● mHHS ↑ 59 ⟶ 85 ● iHOT‐12 ↑ 60 ⟶ 85 ● VAS ↓ 7 ⟶ 2 ● 12% complications (hematoma, hypoesthesia, dysesthesia)

**Open surgery treatment**				

1.	Hogan et al., 2020 [3]	Case series	3 PS patients; minimally invasive release	● Immediate relief, 2 symptom‐free ● No complications

2.	Han et al., 2017 [1]	Retrospective cohort	239 PS; 12 surgery after failed conservative	● VAS reduced ● 83% success ● No complications ● Persistent buttock pain after surgery (*n* = 3)

3.	Son, 2024 [4]	Retrospective cohort	81 PS; decompression ± ligament resection	61.7% success, better with resection, 9.9% recurrence

Across the endoscopic studies, postoperative outcomes consistently showed substantial improvement in pain and function. Most studies reported marked reductions in VAS scores together with increases in mHHS and other hip‐specific functional measures, although the magnitude of improvement varied across cohorts (Tables 1 and 2).

**TABLE 2:** MHHS and VAS outcome.

	**Author, year**	**Study population**		**MHHS**		**VAS**	
		**Male**	**Female**	**Pre-treatment**	**Post-treatment**	**Pre-treatment**	**Post-treatment**
**Endoscopy treatment**							
1.	Martin et al., 2011 [8]	7	28	54.4 ± 13.1	78.0 ± 14.1	6.9 ± 2	2.4 ± 2.6
2.	Park et al., 2016 [12]	33	27	81.7 ± 9.6	91.8 ± 7.6	7.4 ± 1.5	2.6 ± 1.5
3.	Parodi et al., 2023 [14]	20	37	59	85	7	2
4.	Sun et al., 2023 [10]	*n* = 30		64.0 ± 7.4	95.0 ± 3.0	5.0 ± 1.5	0.5 ± 0.7
5.	Aguilera‐Bohorquez et al., 2018 [11]	5	36	52 ± 9,86	78 ± 14.1	13.7 ± 4.4	6 ± 5.9
6.	Quesada‐Jimenez et al., 2025 [9]	5	13	56.5 ± 14.8	82.2 ± 38.2	5.8 ± 1.6	2.5 ± 2.0
7.	Ilizaliturri et al., 2018 [6]	10	5	46.8 ± 13.2	84.9 ± 4.7	7.4 ± 0.7	1.86 ± 0.83
8.	Venermen and Van Melkebeek, 2021 [7]			7.4 ± 0.81	2.2 ± 2.6		

**Open surgery**							

1.	Han et al., 2017 [1]	4	8			9.00 ± 0.91	4.00 ± 2.00

The evidence for open surgery was more limited and less uniform. Available studies suggested postoperative pain reduction and acceptable clinical success in selected patients after failed conservative treatment; however, functional outcomes were less consistently reported, and recurrence was noted in some series (Table 1).

**FIGURE 2:**
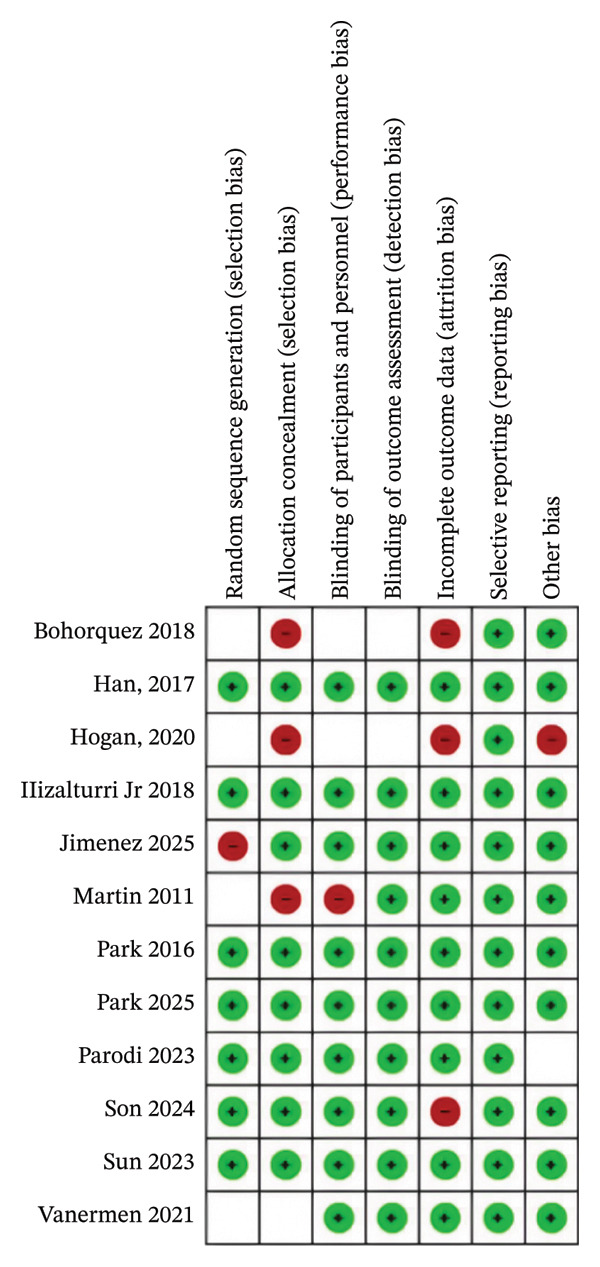
Cochrane risk of bias summary for included studies.

Based on the risk‐of‐bias assessment, the included studies showed mixed methodological quality (Figure 2). Several studies were judged to have low risk in selected domains, whereas concerns remained in relation to sequence generation, allocation concealment, and incomplete outcome data. These limitations should be considered when interpreting the pooled and narrative findings [3, 4, 14].

These findings were crucial as they indicated methodological weaknesses that could affect the overall validity of their systematic review’s conclusions.

Complication rates after endoscopic surgery ranged from 0% to 12%, with most events being minor, including wound infection, hematoma, and transient sensory disturbance. Vanermen and Van Melkebeek reported three wound infections, whereas Parodi et al. reported a 12% complication rate without long‐term sequelae [7, 14]. Separately, Park et al. reported that endoscopic piriformis release was associated with lower 6‐month pain scores than pulsed radiofrequency in a propensity‐matched cohort, although the endoscopic procedure was associated with a somewhat higher complication rate. Because this comparison involved a different comparator and study design from the pooled surgical studies, it was considered supportive qualitative evidence rather than part of the quantitative synthesis [2].

**FIGURE 3:**
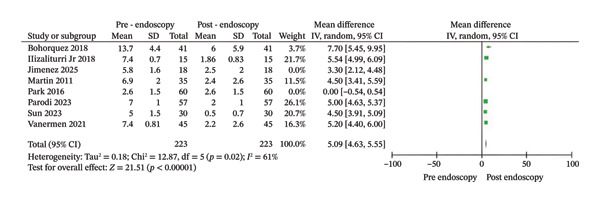
Forest plot of pooled VAS change (endoscopic surgery).

For open surgery, the available evidence was more limited. Han et al. [1] reported an 83% success rate in 12 patients after failed conservative therapy, whereas Hogan et al. [3] described immediate postoperative pain relief without complications in three cases. Son [4] reported a 61.7% success rate, with better outcomes when ligament resection was performed, although recurrence was observed in 9.9% of patients. Overall, open procedures appeared to provide postoperative improvement in selected patients, but the evidence base was limited by inconsistent functional reporting, smaller operative samples, and occasional recurrence (Figure 3).

**FIGURE 4:**
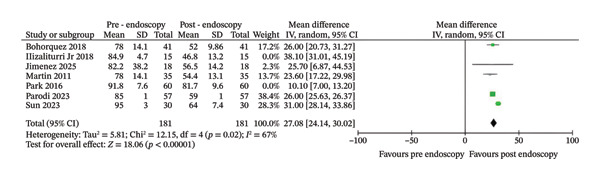
Forest plot of pooled mHHS change (endoscopic surgery).

**FIGURE 5:**
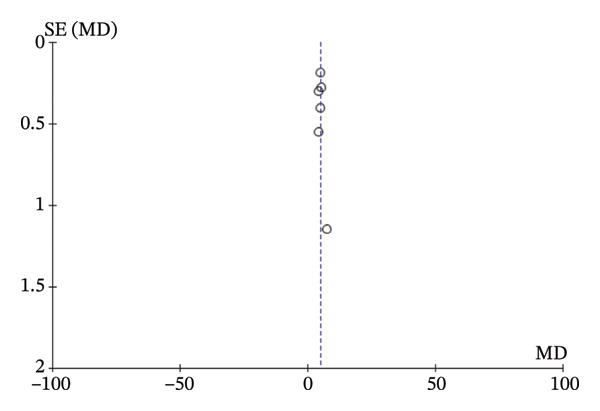
Funnel plot for VAS (endoscopic surgery).

**FIGURE 6:**
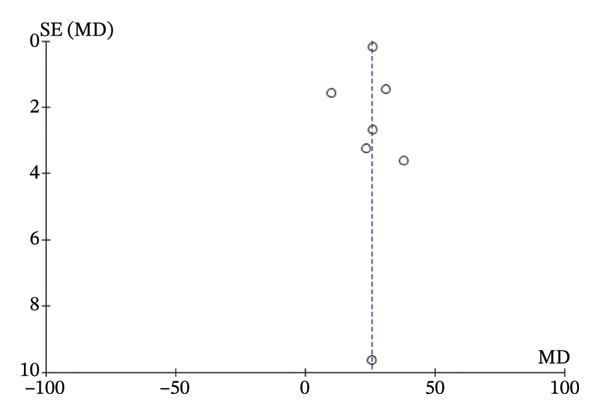
Funnel plot for mHHS (endoscopic surgery).

Meta‐analysis was performed only for endoscopic studies with sufficient preoperative and postoperative numerical data. Six endoscopic studies were pooled for VAS, demonstrating a significant mean reduction of −4.7 (95% CI −5.2–−4.2), indicating a substantial analgesic effect (Figure 3). Heterogeneity was moderate to high (*I* ^2^ = 61%), suggesting variability in baseline severity, surgical technique, and patient selection. For mHHS, six endoscopic studies were pooled and showed a mean improvement of +25.6 (95% CI +16.0–+35.3) (Figure 4). Heterogeneity was high (*I* ^2^ = 97.5%), and sensitivity analysis excluding Quesada‐Jimenez et al. [9] yielded similar pooled estimates with persistently high heterogeneity, suggesting structural differences across studies rather than a single outlier effect. Funnel plots for VAS and mHHS are presented in Figures 5 and 6, respectively.

The funnel plot for VAS showed broad but relatively symmetrical scatter, suggesting no clear evidence of major publication bias (Figure 5). The funnel plot for mHHS showed a relatively symmetrical distribution, although interpretation remains limited by the small number of included studies (Figure 6). Taken together, the available evidence suggests that endoscopic decompression is associated with meaningful postoperative improvement in pain and function in patients with refractory PS. No formal pooled comparison with open surgery could be performed; the present findings do not allow a definitive conclusion that endoscopic surgery is superior or should be considered the preferred approach over open surgery.

Taken together, the available evidence suggests that endoscopic decompression is associated with meaningful postoperative improvement in pain and function in patients with refractory PS. However, because no formal pooled comparison with open surgery could be performed, the present findings do not allow a definitive conclusion that endoscopic surgery is superior or should be considered the preferred approach over open surgery.

## 4. Discussion

This systematic review and meta‐analysis showed that endoscopic decompression was associated with meaningful postoperative improvement in pain and hip‐related function in patients with refractory PS. Pooled analysis of six endoscopic studies demonstrated a significant reduction in VAS and a significant increase in mHHS, although heterogeneity remained high. Complications after endoscopic surgery were generally infrequent and mostly minor. In contrast, the evidence for open surgery was limited, more heterogeneous, and insufficient for pooled quantitative analysis. Therefore, the current findings support the promise of endoscopic decompression, while definitive superiority over open surgery cannot be established from the present data.

The pooled endoscopic data suggest clinically meaningful postoperative improvement, with a mean VAS reduction of −4.7 and a mean mHHS increase of +25.6 [2, 6–9, 12]. These findings are encouraging, particularly because reported complications were generally minor. However, the substantial heterogeneity across studies indicates important differences in patient selection, operative technique, and outcome reporting. Accordingly, these results should be interpreted as supportive of endoscopic decompression rather than conclusive evidence that it is the preferred surgical approach for refractory PS [15, 16].

Martin et al. reported postoperative improvement in pain and function after endoscopic sciatic nerve decompression in patients with deep gluteal syndrome/sciatic nerve entrapment [8]. However, the intervention in that study was not limited to isolated piriformis release and instead addressed several potential sites of sciatic nerve entrapment. Therefore, this study should be interpreted as supporting endoscopic sciatic nerve decompression in deep gluteal syndrome rather than specifically demonstrating the efficacy of isolated endoscopic piriformis release [17–19] Park et al. also reported postoperative improvement in pain and hip‐related function after endoscopic decompression, with better outcomes in atraumatic cases [12]. This finding suggests that underlying etiology may influence postoperative recovery. Separately, Park et al. [2] reported lower 6‐month pain scores after endoscopic piriformis release than after pulsed radiofrequency in a propensity‐matched cohort. Because that study compared surgery with a nonsurgical intervention rather than with open surgery, it should be interpreted as supportive qualitative evidence only and not as evidence of superiority over other surgical approaches [15, 16, 20].

Parodi et al. and Sun et al. also reported favorable postoperative outcomes after endoscopic treatment, supporting the overall pattern of improvement observed across the included endoscopic series [9, 10, 21]. However, these studies should be interpreted within the context of observational designs and heterogeneous outcome reporting [1, 3, 4]. Aguilera‐Bohorquez et al. should be described more cautiously. That study reported clinical improvement after endoscopic treatment using WOMAC and VAIL‐based outcomes in patients who often had associated pathologies and underwent additional procedures [11]. Its findings support postoperative improvement after endoscopic management, but the observed benefit cannot be attributed solely to isolated endoscopic sciatic nerve release or directly compared with open surgery on the basis of VAS and mHHS values.

Quesada‐Jimenez et al. reported postoperative improvement in both pain and functional scores after endoscopic piriformis release with sciatic neurolysis. These findings are consistent with the broader direction of the pooled endoscopic results, but the study does not permit conclusions regarding patient preference, comparison with open surgery, or differential effectiveness according to fibrosis severity [9]. Ilizaliturri reported favorable postoperative outcomes after endoscopic treatment, but the study does not support sex‐based interpretation or speculation regarding anatomical factors favoring minimally invasive surgery [6].

Vanermen and Van Melkebeek also reported postoperative pain improvement after endoscopic treatment, with mostly minor complications [7]. The available endoscopic literature suggests a relatively low complication burden, although cross‐study comparisons should be interpreted cautiously because definitions of complications and reporting practices were not uniform. Comparative statements based on external studies should therefore remain descriptive rather than definitive [22]^.^

Emerging image‐guided and regenerative interventions may expand future treatment options for refractory PS. However, these approaches remain outside the scope of the present pooled surgical analysis and should be considered exploratory at this stage [23–25]. These regenerative strategies aimed to promote tissue repair and nerve modulation, potentially enhancing procedural success rates. Furthermore, recent research had endorsed the integration of advanced imaging modalities, including high‐resolution ultrasound or MRI‐guided procedures, to allow better precision and efficacy of interventions [13, 26, 27].

The evidence for open surgery remained limited and was not sufficient for pooled quantitative synthesis. Han et al. reported postoperative pain improvement and an 83% success rate in surgically treated patients after failed conservative treatment, but functional outcomes were incompletely reported [1]. Son (2024) also suggested that open decompression may provide clinical benefit in selected patients, although recurrence was observed in a subset of cases [4]. Younus et al. should be cited more cautiously, as that paper describes a minimally invasive open approach rather than an endoscopic procedure, and as a single case report, it cannot support broad conclusions regarding the overall role, recovery profile, or complication burden of open surgery [11, 28–30].

The substantial heterogeneity observed in the meta‐analysis likely reflected variation in clinical settings, surgical techniques, baseline severity, and outcome reporting across studies [31]. This heterogeneity limits the certainty of pooled estimates and reinforces the need for cautious interpretation. Nevertheless, the overall direction of effect across the endoscopic studies was consistently favorable [7, 9].

**FIGURE 7:**
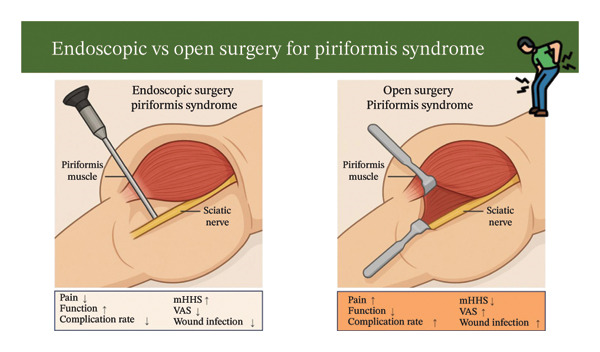
Summary of endoscopic vs. open surgery for piriformis syndrome.

Figure 7 provides a schematic summary of the overall findings of this review. In general, the available evidence suggests that endoscopic surgery is associated with postoperative improvement in pain and function and with mostly minor complications. The evidence for open surgery also suggests potential clinical benefit in selected patients, but it remains limited and less consistently reported. Therefore, Figure 7 should be interpreted as an illustrative summary rather than as proof of definitive superiority of one surgical approach over the other [32].

Despite encouraging findings for endoscopic treatment, the open‐surgery literature in this review was limited by small operative samples, inconsistent functional reporting, and insufficient quantitative data. These limitations prevented a robust comparative synthesis between endoscopic and open procedures. Future studies should standardize diagnostic criteria, surgical techniques, and outcome measures to enable more reliable comparative evaluation.

## 5. Conclusion

In conclusion, the available evidence suggests that endoscopic decompression appears to provide favorable outcomes in carefully selected patients with refractory PS, particularly in terms of pain relief and functional improvement. However, definitive superiority over open surgery cannot be established from the current literature because direct comparative studies are lacking and the available open‐surgery data remain limited. Further well‐designed comparative studies are needed to clarify the relative effectiveness of endoscopic and open surgical approaches in the management of PS.

## Author Contributions

Fanny Indra Warman: conceptualization; methodology; data curation; formal analysis; writing original draft; and project administration.

Jifaldi Afrian Maharaja Dinda Sedar: investigation; data curation; and writing review and editing.

Muhammad Hanun Mahyuddin: methodology; validation; visualization; and writing review and editing.

Williem Harvey: supervision; resources; and critical revision of the manuscript.

## Funding

No funding was received for this manuscript.

## Conflicts of Interest

The authors declare no conflicts of interest.

## Data Availability

The data that support the findings of this study are available from the corresponding author upon reasonable request.
